# Coupling instantaneous energy-budget models and behavioural mode analysis to estimate optimal foraging strategy: an example with wandering albatrosses

**DOI:** 10.1186/2051-3933-2-8

**Published:** 2014-04-23

**Authors:** Maite Louzao, Thorsten Wiegand, Frederic Bartumeus, Henri Weimerskirch

**Affiliations:** UFZ-Helmholtz Centre for Environmental Research, Permoserstrasse 15, 04318 Leipzig, Germany; Centre d’Etudes Biologiques de Chizé, UMR 7372 CNRS – Université de la Rochelle, 79369 Villiers en Bois, France; Instituto Español de Oceanografía, CO Xixón, Camín de l‘Arbeyal s/n, Xixón, 33212 Spain; ICREA-Movement Ecology Laboratory (CEAB-CSIC), Accés Cala St. Francesc 14, 17300 Blanes, Spain; CREAF, Cerdanyola del Vallès, Barcelona, 08193 Spain

**Keywords:** Optimal foraging theory, Energy-budget model, Behavioural clustering, Oceanic winds, Oceanic predator

## Abstract

**Background:**

How foragers move across the landscape to search for resources and obtain energy is a central issue in ecology. Direct energetic quantification of animal movements allows for testing optimal foraging theory predictions which assumes that animals forage so as to maximise net energy gain. Thanks to biologging advances, we coupled instantaneous energy-budget models and behavioural mode analysis to test optimal foraging theory predictions on wandering albatross *Diomedea exulans* during the brooding period. Specifically, the instantaneous energy-budget model considered the energetic balance (i.e., the difference between empirical energy gain data and modelled energy expenditure via heart rate values) along the trajectory of a given individual. Four stereotypic instantaneous behavioural modes were identified based on trajectory properties (e.g., speed and turning angle) by applying a new algorithm called Expectation Maximization Binary Clustering. Previous studies on this species have shown that foraging-in-flight is the optimal foraging strategy during the incubation period when albatrosses undertake long-distance movements but no specific foraging strategy has been determined for shorter foraging movements (e.g., brooding period).

**Results:**

The output of our energy-budget model (measured as net energy gain) highlighted the potential optimality of alternative search strategies (e.g., sit-and-wait) during brooding, when birds may be subjected to specific energetic trade-offs and have to adapt their foraging strategies accordingly. However, not all birds showed this pattern, revealing the importance of considering individual variability in foraging strategies, as well as any switching among strategies, before drawing population-level generalizations. Finally, our study unveils the importance of considering fine scale activities to make realistic estimates of trip energy expenditure for flying birds at sea.

**Conclusions:**

The up-scaling of accurately measured fine-scale energy patterns is essential to quantify energy balances, and their fluctuations by season of different activities among individuals or populations. In particular, we offer new insights for the energetic quantification of the effect of changing oceanic winds on the biology of pelagic predators in the southern oceans.

**Electronic supplementary material:**

The online version of this article (doi:10.1186/2051-3933-2-8) contains supplementary material, which is available to authorized users.

## Background

Free-ranging animals have to adapt their search movements and foraging strategies to current environmental conditions in order to fully cover their energetic requirements for reproduction and survival [1]. Thus, the ecological energetics of animals is essential to link movement behaviour to different population-level processes. Foraging is a complex process where both extrinsic and intrinsic factors play an important role and are intimately interlinked [2]. On the one hand, the internal state (i.e., physiology that drives the organism to fulfil one or more goals such as searching for food) governs the decision of foraging destinations (i.e., where and when to move, [3]). On the other hand, external environmental conditions such as prey availability and forcing factors (e.g., wind) constrain decision-making [3]. This outlines the importance of quantifying energetic balances (i.e., net energy gain–the difference between energy gain and energy expended) to test optimal foraging theory predictions which assume that animals should forage in a way that maximises net energy gain [1].

A detailed knowledge of the ecological energetics of free-ranging animals is essential for understanding their spatial distribution patterns and identifying key parameters affecting the movement process [4–6]. Such information is especially interesting for research in foraging ecology [7], but also in evolutionary ecology and conservation [8–10]. Ongoing development in biologging techniques have substantially improved our understanding of movement ecology of free-ranging animals in the last two decades [9], providing simultaneously information about the movement, energy expenditure and behaviour of the monitored individual, as well as contemporaneous environmental conditions [6]. This progress has paved the way for a unified comprehensive movement paradigm called *Movement Ecology* sustaining a multidisciplinary integration of existing scientific disciplines (e.g., behavioural, spatial, computational and quantitative ecology) [3, 6].

The open ocean represents a highly dynamic environment where resource distribution varies in space and time over a wide range of scales [11]. In the open ocean, wind is an important forcing factor for pelagic seabirds (e.g., Procellariiformes) as an energy source for their movements [12–14]. Thus, they must adapt their movements to wind conditions at multiple spatiotemporal scales from small-scale local conditions to large-scale weather systems [14, 15]. The southern oceans show the strongest wind conditions worldwide and foraging in this environment is challenging. Wide-ranging seabirds are adapted to these harsh conditions, and are able to reduce energy costs in flight by using wind in an optimal way [13]. During these flights, they spend little energy when searching for prey thanks to dynamic soaring, especially when cruising with favourable winds [12, 13, 16]. Clearly, low foraging costs are a critical component of the unique life-history pattern of some pelagic birds that face strong energy constraints imposed by large distances between breeding and foraging grounds [13]. In addition, energetic constraints vary depending on breeding stage with chick-rearing being energetically the most demanding period [17].

The aim of our study is to make an estimation of the fine-scale ecological energetics of a free-ranging predator to test a central hypothesis in optimal foraging theory which states that animals should forage so as to maximise net energy gain. To do so we quantified the ecological energetics of individual animals by coupling instantaneous energy-budget models with behavioural modes analyses. We developed a fine-scale energy-budget model considering the energetic balance along the trajectory of a given individual, including both energy gain and expenditure. Energy gain was computed by means of empirical prey capture data, whereas energy expenditure was modelled based on empirical heart rate values. In addition, we used a novel algorithm: the Expectation-Maximization Binary Clustering (EMBC) [18] to behaviourally annotate animal trajectories based on speed and turn estimations from successive pairs of locations. The EMBC algorithm fills a gap in movement trajectory segmentation procedures (others such as tortuosity [19], first-passage time [20], residence time [21], and positional entropy [22]) because it (i) minimizes the need for supervision, (ii) limits analytical complexity (number of parameters and prior assumptions), (iii) avoids sensitivity to prior assumptions and/or initial values, and (iv) captures sufficiently general and biologically meaningful semantics [18]. The present study is an effort to demonstrate the importance of integrating instantaneous energy budgets within the movement ecology paradigm to fully understand how foragers moves across the landscape, drawing special attention to individual-level decisions.

## Methods

### Animal model

We studied wind-dependent wandering albatross *Diomedea exulans* using data from long-term tracking programmes in the Southern Indian Ocean [9] where the foraging strategy of this species have been well characterised [23–26]. When they make long-distance movements, during the incubation period, birds travel constantly and quickly to maximize their probability of encountering isolated prey or prey patches using the foraging-in-flight (FII) search strategy. In addition, birds are also attracted to oceanographic features such as shelf-breaks and seamounts where they spend more time searching for prey, using an area-restricted-search (ARS) behaviour. Even when sitting on the water, wandering albatrosses actively search and catch prey by showing a sit-and-wait (SAW) foraging strategy. During the brooding period, birds make shorter foraging trips and in this study we explicitly compared net energy gain to assess assumptions of optimal foraging theory during this stage.

### Conceptual energetic framework

We built an instantaneous time-energy budget model along the track of individual albatrosses based on the simple assumption that net energy gain is the balance between energy gain and energy expenditure:
1

**Figure 1:**
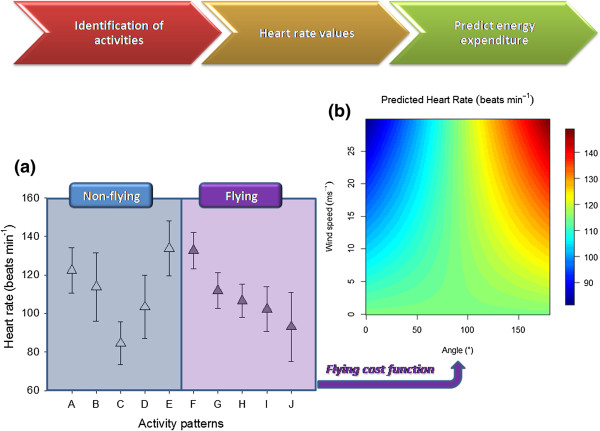
**Workflow of the estimation of heart rate values depending on the activity. (a)** Mean (± SD) values of heart rate (beats min^-1^) for different activities (extracted from Figure 2 in [28]). Non-flying modes were from A to E (A: ‘landing’; B: ’30 min after landing’; C: ‘resting’; D:’30 min before take-off’, E: ‘take-off’; represented by white triangles). Flying modes were from F to J (F,G, H, I and J correspond to ‘flying’10,30,60,120 and 720 min after take-off; represented by dark grey triangles). **(b)** Flying cost model based on [13, 14]. Estimated values of energy expenditure values while flying are obtained in heart rate units (beats min^-1^) considering the effect of the angle between flight track and wind direction *θ* and wind speed *w. w* ranges from 0 to 30 m s^-1^, whereas *θ* ranges from 0° to 180° indicating that birds were flying with tail and head winds, respectively. The cost of flying was intermediate in two situations: in the absence of wind and when birds were flying with cross winds (light blue values).

Energy gain was estimated based on (1) empirical prey capture data that provided instantaneous mass intake at high temporal resolution [25] and (2) conversion factors considering the diet of wandering albatrosses (i.e., the energetic content and proportion of squid and fish in the diet) [27]. Energy expenditure was estimated by developing an instantaneous energy expenditure model to obtain a continuous measure of heart rate values during a foraging trip, by (1) identifying activity patterns in detail, (2) estimating heart rate values of each activity, including a cost function for flying, and (3) using a non-linear relationship between heart rate and oxygen consumption to transform heart rate values to energy expenditure estimations (see Figure 1). Our energy expenditure predictions are equivalent to field metabolic rates and explicitly consider different non-flying (‘landing’, ‘30 min after landing’, ‘resting’; ‘30 min before take-off’ and ‘take-off’) and flying activities (‘flying’ 10, 30, 60, 120 and 720 min after take-off) that are known to have different energetic costs [13], in addition to implicitly including other processes related to somatic maintenance such as thermoregulation. Estimates of energy expenditure of those processes are not currently available and this information could be included in the model when more detailed information on the ecological energetics of pelagic birds become available.

The energy-budget model predictions’ were validated against an independent empirical distribution of observed body mass change between the end and start of foraging trips. It was validated under the assumption that trip net energy gain (TNEG) converted to mass units should roughly correspond to albatross body mass (BM) change (i.e., difference between body mass at departure for the sea and at return on the nest), considering that 1 g of albatross fat is equivalent to 19.8 kJ (i.e., conversion factor) [29]. This relationship is exemplified in the following equation:
2

Given the short duration of foraging trips during brooding (mean: 3 days, range: 0.2–12; [30]), we assumed that body mass difference is an approximation to prey intake that will be provided to chicks and in turn we considered that there was no assimilation. Thus, we assumed that foraging energy expenditure was obtained almost exclusively from their energetic reserves and that birds did not likely obtain energy from ingested prey. This observation is realistic for the 30-day brooding period since this period is the only portion of the annual cycle when wandering albatrosses undergo a significant decrease in body mass, suggesting that they cannot meet their energy requirement [27, 28, 31].

### Empirical data

We developed and validated the energy budget model using GPS tracking data of 45 wandering albatrosses during the brooding period of 2002–2005 [25]. Birds were fitted simultaneously with a GPS (providing location coordinates within 5 m every 10 s; New Behaviour, Zurich), a stomach temperature transmitter and associated receiver-recorders (Wildlife Computer, Redmond, WA). Albatrosses were induced to swallow a 20-g pill which transmits stomach temperature every 15 s to a receiver/logger attached to the back of the bird. The changes in temperature allow estimation of the timing of prey ingestion and the mass of prey capture along the track (more details in [25]). The total mass of the equipment was 90 g (0.7%–1.2% of body mass) which is well below the recommended 3% threshold [32]. At departure and return to the nest albatrosses were weighted (without equipment) to the nearest 50 g using a Salter spring balance (Salter Weightronix Ltd, West Bromwich, UK).

Due to several logistic constraints (i.e., electronic problems, premature loss of logger), prey capture data were available only for 18 foraging trips. Because not all foraging trips recorded simultaneously geographic position and prey capture data, we obtained 5 completely tracked foraging trips, 5 near-complete (tracks stopped recording when birds were heading to the colony), and 8 incomplete. Available tracking data corresponded to a 4-year period and we did not find inter-annual differences in foraging trip characteristics such as mean flying speed (*F*_*3,14*_ = 0.38, *P* = 0.766), maximum flying speed (*F*_*3,14*_ = 1.15, *P* = 0.364) and distance travelled per day (*F*_*3,14*_ = 0.46, *P* = 0.715). Thus, all years were pooled to develop the instantaneous time-energy budget model. Before any data processing, locations obtained from GPS with an associated speed between successive positions above 90 km h^-1^ were discarded [33].

Estimation of energy expenditure was based on mean and SD values of heart rate obtained empirically when wandering albatrosses were equipped with miniaturized external heart-rate recorders (PE4000, Polar, Elektro Oy, Kempele, Finland), satellite transmitters (Microwave Telemetry, Columbia, MD, USA) and activity recorders (Francis Instrument, Cambridge, UK) [13] (Figure 1). GPS foraging trips were resampled to obtain one position every 1 min in order to match the temporal unit of heart rate values (beats min^-1^) [13]. For each position, we estimated the distance from the previous location and to the colony, travel speed, flight direction (angle with respect to north), the azimuth and elevation of the sun (for estimating the day/night periods), as well as wind direction *α,* wind speed *w* and the angle between albatross flight direction and wind direction *θ.* More information on wind data can be found in the Additional file 1. For the diel cycle, we defined ‘night’ as the period in which the sun was six degrees or more below the horizon and ‘day’ otherwise using the ‘tripEstimation’ package [34]. We estimated flight direction (i.e., angle with respect to north) using the ‘circular’ package [35].

### Estimation of net energy gain

Instantaneous net energy gain was estimated at our basic temporal unit (i.e., 1 min) as the difference between energy gain and energy expenditure based on Eq. 1. Similarly, total trip net energy gain was estimated by cumulative summing instantaneous net energy gain along the foraging trip.

Regarding the estimation of instantaneous energy gain, we first estimated instantaneous mass intake (kg min^-1^) based on prey capture data. Then instantaneous mass intake was transformed into energy gain by assuming that the 75% and 25% of prey capture data corresponded to squid and fish (prey identification was not possible), with an energetic content of 5.61 kJ g^-1^ and 4.64 kJ g^-1^, respectively [29, 36].

Regarding estimation of instantaneous energy expenditure (R-based script will be made available on request to the corresponding author), we first identified albatross activities at the instantaneous level along the foraging trip (see workflow in Figure 1). The two main activities (i.e., flying and sitting on the water) were identified based on the travel speed by using the threshold of 10 km h^-1^. Those locations with a travel speed above and below 10 km h^-1^ corresponded to flying and resting activities, respectively [37]. Detailed tracking data showed that landing and take-off were characterised by elevated heart rate values, as well as those periods (i.e., 30 min) preceding and following landing and take-off [13]. Therefore, we considered five non-flying activities (A: ‘landing’, B:’30 min after landing’, C: ‘resting’; D: ‘30 min before take-off’ and E: ‘take-off’) and five flying activities (‘flying’ 10, 30, 60, 120 and 720 min after take-off, corresponding to F, G, H, I and J activities) as described in [13] (see an example in Additional file 2).

While energy expenditure during flight is usually considered when analysing optimal flying pathways of pelagic seabirds in relation to wind conditions, energetic cost of resting (i.e., sitting on the water), take-off and landing have been seldom considered (e.g., [14, 38]). However, we included the energetic cost of resting in our energy balance because albatrosses can spend on average 46.7% of their time on water (range: 24.6–68.3%, present study) whereas this percentage was higher during the night (average: 63.5%, range: 37.1-95.1%) compared to the day (average: 28.5%, range: 10.2–61.8%) and because the energetic expenditure of resting is nearly as costly as flying with favourable wind conditions [13]. One limitation of our approach was that we were not able to provide different heart rate values for resting and sitting on the water while trying to locate, secure and swallow prey, provided that the latter provide higher heart rate values.

The effect of wind speed *w* on energy expenditure cannot be neglected since wind speed can have important implications for the energy budget during flying activities [14, 38]. To account for the impact of *w* and the angle between flight and wind direction *θ* on energy expenditure, we adapted the flying cost function developed by [14] to wandering albatrosses using field data from [13]. The flying cost function was applied to flying activities (F to J in Figure 1a) in order to obtain energy expenditure values (i.e., heart rate; more details of flying cost development is provided in Additional file 3) (Figure 1b). Our flying cost function was valid since the relationship between the angle between flight and wind direction and energy expenditure patterns during flight were similar in both studies, even though they were based in different energy expenditure approaches (Additional file 3) [13, 14]. Thus, the flying cost model was able to provide energy expenditure estimates based on two variables: wind speed *w* (ranging from 0 to 30 m s^-1^) and the angle between flight and wind direction *θ* (ranging from 0° to 180°, indicating that birds were flying with tail and head winds, respectively). The energy expenditure while flying was intermediate in two situations: in the absence of wind and when birds were flying with cross winds (light blue values in Figure 1b). From this intermediate reference level, energy expenditure decreased when birds were flying from cross winds to tail winds at increasing wind speed. On the contrary, energy expenditure increased when birds were flying from cross winds to head winds at increasing wind speed (Figure 1b).

Instantaneous heart rate values at 1-min resolution were converted to energy expenditure values based on the relationship between heart rate and oxygen consumption. This relationship was linear for incubating (resting) wandering albatrosses [39], but could follow a power-curve when birds are engaged in locomotory activities (i.e., larger oxygen pulse conditions) [40]. In fact, when other flying birds (e.g., wild geese) were active in a wind tunnel the relationship between heart rate and oxygen consumption was significantly different between walking and flying (i.e., power-curve relationship [41]). This was also true for black-browed albatrosses *Thalassarche melanophrys* walking on a treadmill [4, 42]. In the present study, we followed the power-curve relationship of black-browed albatrosses and included both basal [39] and maximum values of heart rate for wandering albatrosses [13] to obtain a mass specific power-curve relationship for the species:

3where *Oxygen consumption* is in mLmin^-1^ kg^-1^.Then, oxygen consumption values were converted to energy units by assuming that 1 mL of oxygen is equivalent of 20.112 J [4]. A mass specific relationship was used since heart rate basal values increase with body mass in wandering albatrosses [39]. We acknowledge that further research would be needed to directly measure heart rate and oxygen consumption on flying wandering albatrosses to improve this relationship (c.f. [41]), although this would be logistically and biologically difficult with present technologies.

### Energy-budget model validation

We validated our energy-budget model (i.e., the assessment of the accuracy of predictions) by calculating the agreement between observed and predicted values. In the present study, we predicted the body mass change of wandering albatrosses by transforming trip net energy gain to mass units applying Eq. 2. The limitation of the present validation approach is that we needed complete foraging trips since body mass change is a reflection of the whole trip net energy gain. In order to robustly validate our modelling approach, we additionally used instantaneous energy expenditure estimates measured with the Doubly Labelled Water method (see below). Thus, we validated our energy budget model at two different temporal scales: at the foraging trip and instantaneous levels.

At the trip level, we used the mass gain measured by the birds equipped with stomach temperature pills and assumed that the trip net energy gain is a measure of albatross’ body mass difference between arrival and departure for the foraging trip, Eq. 2. Predictions of body mass change were also contrasted against an empirical distribution from the long-term tracking database of wandering albatross [9, 43] for 97 independent individual (more details in Additional file 4).

Regarding validation at the instantaneous level, we contrasted our predictions of energy expenditure (based on heart rate values) against an empirical distribution of energy expenditure of wandering albatrosses measured by a different method (Doubly Labelled Water, DLW) [44–46]. DLW provides energy expenditure estimates averaged over the measurement period which is normally restricted to a few days [46]. In contrast, heart rate provides continuous measurement that can be used to estimate energy expenditure of specific activities [13]. In order to pool DLW-based available data, we ensured (by means of ANOVA) that there were no differences between years (season 1982/1983 vs. 1998 for brooding: *F*_*1,18*_ *= 0.885*, *P = 0.359* and breeding stages (for 1998: *F*_*1,17*_ *= 0.021*, *P = 0.886*). We expect that our heart rate (HR)-based estimates of energy expenditure for free-ranging albatrosses might be lower than DLW-based estimates [47]. One of the best approaches to accurately validate predictions using heart rate methods is to simultaneously measure energy expenditure with HR and DLW in the same individual while foraging at sea. However, this has not yet been done in the field [40, 48] showed that energy expenditure measurements of incubating wanderers did not significantly differ when using DLW or HR methods, even if the latter provided lower estimates. However, when marine predators are engaged in energetically more costly activities at sea DLW-based energy expenditure values could be overestimated due to technique assumptions [47], providing higher values of energy expenditure than HR methods. These authors also suggest the possibility that HR methods could underestimate energy expenditure values [47].

Finally, we compared the modelling output including ten detailed (fine-scale) activities (*sensu*[13]) with estimations under the common practice of using only the two wide-scale activities: resting and flying (e.g. [49]). For that, we regrouped fine-scale activities from A to E as resting and from F to J as flying.

### Coupling instantaneous energy-budget models and behavioural mode analysis

By coupling instantaneous energy-budget models with behavioural mode analysis, we were able to provide an energetic perspective to the characterisation of foraging strategies. We applied a new algorithm called Expectation Maximization Binary Clustering (EMBC) to obtain behavioural modes from direct analysis of movement trajectories. The EMBC algorithm fills a gap in movement trajectory segmentation procedures by reaching a good compromise between meaningful and easily interpretable behavioural segmentation and sound (and robust) statistical performance [18]. As an unsupervised and non-intensive computing method, the EMBC algorithm is particularly suited for big data and large scale analyses where comparisons across species, sampling schemes, tracking technologies, and ecological contexts are looked for [18].

The EMBC algorithm models behavioural modes as a multivariate Gaussian mixture [18]. Here we considered the simplest behavioural space possible defined by two movement variables: speed and turning angle. The EMBC algorithm determines the maximum likelihood partition into four regions characterized by high and low values of each variable, in this case, for speed/turn values. This partitioned space can be then associated to stereotypic behaviours such as relocation (e.g., high speeds and low turns), extensive search (e.g., high speeds and high turns), intensive search (e.g., low speeds and high turns) and as resting (e.g., low speeds and low turn) [18]. Intensive search mode is referred to negligible horizontal displacement (e.g., low speed) with active (e.g., high turning angle) searching behaviour. Thus, the EMBC algorithm classified each position with an instantaneous behavioural mode at 1-min resolution. Then, individual foraging strategies were defined by the percentages of these four stereotypic modes within the foraging trip and help identifying the main foraging strategy used by each bird, as well as secondary alternative strategies. According to previously described foraging strategies, those individuals showing high percentages of relocation, extensive search, intensive search and resting would be using foraging-in-flight (FII), area-restricted search (ARS), sit-and-wait (SAW) and resting (RES) strategies, respectively.

In order to group individual foraging strategies, we performed a hierarchical clustering analysis based on the relative duration (%) of each behavioural mode within a foraging trip using the *Pvclust* package, specifying the Euclidean distance and Ward agglomeration method [50]. *Pvclust* calculates *P*-values for hierarchical clustering via multiscale bootstrap resampling and significant clusters with probability *P* ≥ 0.95 were extracted. After individual grouping, clusters were characterised by means of movement and energetic parameters. Movement parameters included both mean and maximum speed (m s^-1^), maximum range (i.e., maximum distance attained from the colony; km) and trip duration (h). Energetic parameters included daily energy expenditure (kJ d^-1^), daily energy gain (kJ d^-1^), daily net energy gain (kJ d^-1^), prey mean weight (g), prey weight variability (i.e., SD in g), number of prey per day, foraging efficiency and mass at departure (kg). Then, an ANOVA analysis (when normally distributed) or a non-parametric Kruskal-Wallis test was applied for selecting the most significant parameters characterising clusters. Significance was set at *P* < 0.1 and marginal significance at *P* < 0.2. Finally, we classified low, intermediate and high mean values of behavioural modes, movement and energetic parameters per foraging strategy to better illustrate clustering output.

## Results

### Energy-budget model validation

**Figure 2:**
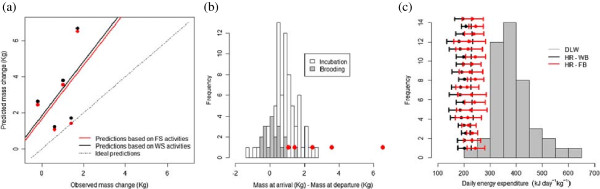
**Energy-budget model validation plots. (a)** Total TNEG converted to mass units (predicted body mass change) should approximate to body mass change between the arrival and departure (observed). 5 complete foraging trips were only available out of 18. Red and black dots represent predicted body mass change based on fine-scale (FS; 10 activities) and wide-scale (WS; only flying and resting) activities, respectively. **(b)** Histogram of the observed distribution of body mass change for incubation and brooding. Predicted values are represented by red dots. **(c)** Individual predictions of energy expenditure (mean and interval confidences) based on heart rate (HR) values corresponding to 18 albatrosses contrasted against an empirical distribution of energy expenditure based on the Doubly Labelled Water (DLW) method (grey histogram) from [44–46]. Individual predictions differed by considering wide-scale (WB in black, only flying and resting) and fine-scale (FB in red,10 activities from A to J, Figure 1) activities.

We validated our energy budget model (Eq. 1) based on fine-scale activities using the five complete foraging trips. Energetic estimations can be found in Table 1. Total prey capture per trip ranged from 2.09 to 7.46 kg which corresponded to a trip energy gain ranging from 10224 to 36414 kJ. Trip energy expenditure estimated via heart rate ranged on average from 4521 to 7601 kJ. Similarly, average trip net energy gain varied from 5199 to 31892 kJ. The observed body mass change varied between -0.2 and 1.7 kg, while our predictions ranged between 1.06 and 6.53 kg (Table 1, Figure 2a). Moreover, trip net energy gain converted to mass units approximated albatross body mass difference between arrival and departure since our predictions fell within the empirical distribution of body mass difference between arrival and departure for brooding (Table 1 and Figure 2b).

**Table 1:** **Summary of relevant energetic output associated to18 complete foraging trips of wandering albatrosses considering both fine- and wide-scale activities**

Foraging trip	Sex	Trip	OBMC (kg)	TPC (kg)	TEG (kJ)	Fine-scale activities			Wide-scale activities		
						TEE (kJ)	TNEG (kJ)	PBMC (kg)	TEE (kJ)	TNEG (kJ)	PBMC (kg)
882	F	Complete	-0.2	3.52	17172	5275	11896	2.44	4342	12829	2.63
075	M	Complete	1	4.89	23875	6505	17370	3.56	5407	18468	3.78
778	M	Complete	1.4	2.98	14555	7601	6956	1.42	6203	8353	1.71
975	M	Complete	1.7	7.46	36414	4521	31892	6.53	3772	32641	6.69
041	M	Complete	0.6	2.09	10224	5024	5199	1.06	4230	5993	1.23
959	F	Incomplete	0.6	2.09	10190	2512	7677	1.57	2197	7992	1.64
837	M	Incomplete	NA	0.88	4287	7298	-3011	-0.62	6521	-2234	-0.46
039	F	Incomplete	1	1.11	5425	693	4731	0.97	636	4788	0.98
053	M	Incomplete	0.8	4.76	23255	2765	20490	4.2	2432	20823	4.26
264	M	Incomplete	0.5	0.94	4604	1682	2922	0.6	1364	3240	0.66
440	M	Incomplete	0.5	3.66	17870	2914	14955	3.06	2411	15459	3.17
471	M	Incomplete	0.7	1.82	8901	1841	7060	1.45	1495	7405	1.52
035	F	Incomplete	0.8	7.26	35457	3403	32053	6.56	3013	32443	6.64
166	M	Incomplete	1	0.65	3178	3962	-783	-0.16	3364	-185	-0.04
167	F	Incomplete	-0.3	3.85	18807	3439	15368	3.15	2943	15864	3.25
306	F	Incomplete	0.6	0.44	2158	1508	650	0.13	1281	877	0.18
708	M	Incomplete	1.6	2.97	14486	2824	11661	2.39	2327	12159	2.49
734	F	Incomplete	0	2.44	11894	2983	8910	1.82	2515	9378	1.92

OBMC: Observed Body Mass Change; TPC: Trip Prey Capture (kg); TEG: Trip Energy Gain (kg), TEE: Trip Energy Expenditure, TNEG: Trip Net Energy Gain; PBMC: Predicted Body Mass Change; F: female. M: male.

At the instantaneous level, we predicted a mean instantaneous energy expenditure estimated via heart rate of 194 kJ kg^-1^ day^-1^ (range: 132 – 234) and 229 kJ kg^-1^ day^-1^ (range: 160 – 288) based on the wide and fine scale (considering 2 and 10) activities for the 18 foraging trips, respectively (confidence intervals in black and red, respectively, see Figure 2c). Overall, fine-scale based energy expenditure predictions were on average 18.0% (range: 8.8 – 23.2) higher than wide-scale based energy expenditure. This is partially due to the importance of considering fine-scale activities since ‘flying 10 min after take-off’ (mean: 346 kJ kg^-1^ day^-1^; range: 343 - 350) and take-off (mean 313 kJ kg^-1^ day^-1^; range: 191- 446) were the energetically most costly activity even if accounting only for less than 1.5% of the at-sea activity (Additional file 5). Predictions fell within the range of observed values (for brooding; Figure 2c) but they were significantly lower (*F*_*1,36*_ *= 53.24*, *P < 0.001*) than energy expenditure measurements based on Doubly Labelled Water method.

### Albatross trajectories

**Figure 3:**
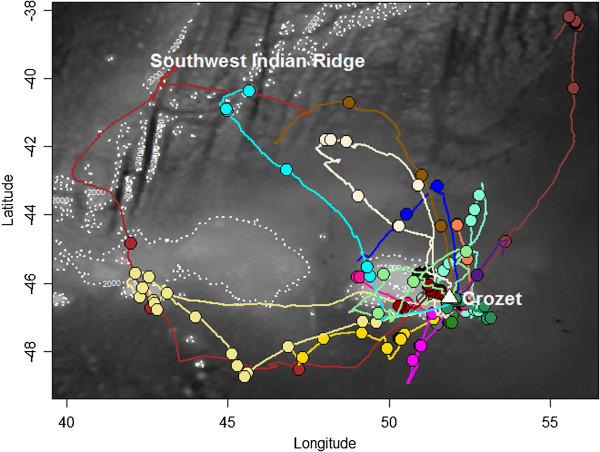
**Map showing all GPS tracks (n = 18) with prey capture information of wandering albatross breeding in Crozet (Southern Indian Ocean), during the brooding of 2002-2005 period.** Lines and points represent continuous trajectories and prey captures, respectively. Each colour represents one individual. The white triangle indicates the breeding colony. Background grey scale corresponds to the bathymetry and isobaths of 200, 1000 and 2000 m are also highlighted (white dotted lines).

**Figure 4:**
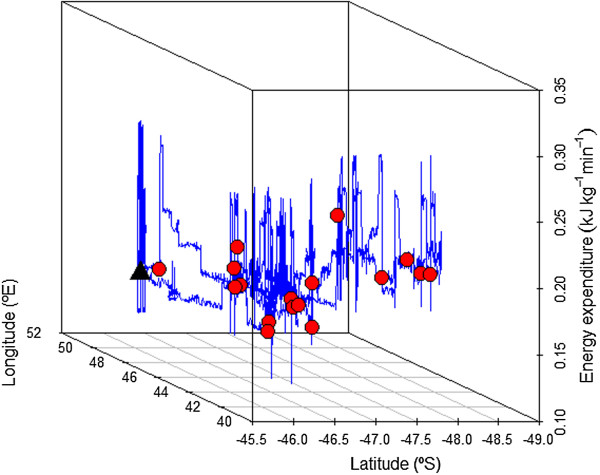
**Spatial illustration of the fine-scale energy expenditure (kJ kg** ^**-1**^ **min** ^**-1**^ **) of individual 075 (prey capture points in red).** The colony is represented by the black triangle.

The 18 tracked albatrosses travelled over the Crozet shelf and surrounding marine areas around their breeding sites, whereas the longest foraging trips headed to the Southwest Indian Ridge (NW of Crozet; Figure 3). All birds explored the western sector of the breeding colony, heading to all possible directions between 180° and 360° N. On average, foraging trips had a maximum foraging of 437.7 km (range: 88.9 – 1004.0) and lasted 42.4 h (range: 5.1 – 81.7). Albatrosses spent on average 46.7% of their time sitting on the water (range: 24.6 – 68.3), whereas this percentage was higher during the night (average: 63.5%, range: 37.1 - 95.1) compared to the day (average: 28.5%, range: 10.2 – 61.8). Figure 4 and Additional file 6 show examples of the typical dual activity pattern: birds actively moving during the day with several landings and take-offs, resulting in higher values of energy expenditure, while travel speed and energy expenditure values were lower during the night.

### An energetic approximation to foraging strategy identification

**Figure 5:**
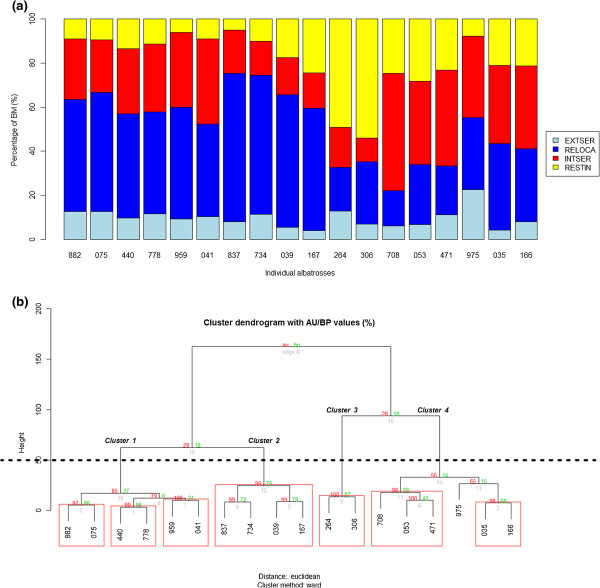
**Identification of foraging strategies based on individual percentages of behavioural modes. (a)** Relative duration (%) of each behavioural mode (EXTSER: extensive search; RELOCA: relocation; INTSER: intensive search; RESTIN: resting) within the 18 individual foraging trips. **(b)** Output of the hierarchical clustering analysis to group individual foraging strategies based on the relative duration (%) of each behavioural mode using the *Pvclust* package via multiscale bootstrap resampling. Significant clusters with probability *P* ≥ 0.95 are indicated by red rectangles, as well as the 50% similarity level by the black dotted line.

The EMBC algorithm classified instantaneous behavioural modes into four clusters (i.e., regions) of high and low values for speed/turn values that were used to build individual ethograms (Additional file 6). The percentage of each behavioural mode greatly varied among individuals (Figure 5) and help defining the main and alternative foraging strategies used along each foraging trip. Hierarchical clustering analysis performed on the individual percentage of behavioural modes identified seven clusters with *P* ≥ 0.95 (indicated by red rectangles, Figure 5b), but we applied the 50% similarity level to obtain population level foraging strategies (clusters 1 to 4, Figure 5b). Our approach identified four clusters which significantly differed first by the percentage of relocation and resting (*P* < 0.1), followed by the percentage of intensive search (Table 2). The percentage of extensive search had no effect on cluster identification since it did not significantly differ among individuals (Table 2, Figures 5a and b can be compared directly since individuals have been accommodated in the same order), and we did not consider it meaningful for further interpretation. Regarding movement parameters, mean travel speed, maximum range and maximum travel speed explained clustering output (Table 2). Regarding energetic parameters, daily energy expenditure, daily net energy gain and daily trip energy gain explained cluster differences (Table 2).

**Table 2:** **Average values of both movement and energetic parameters characterising each cluster, ordered by increasing** ***P*** **value**

Type	Variable name	Normality	Statistic	***P*** value	Cluster 1 FII/SAW mean (± sd)	Cluster 2 FII mean (± sd)	Cluster 3 RES mean (± sd)	Cluster 4 SAW mean (± sd)
**Movement**	Mean speed (m s^-1^)	Yes	13.01	<0.001,*	7.76 (± 1.03)	9.91 (± 1.37)	4.6 (± 0.82)	4.58 (± 1.55)
**Behavioural**	RELOCA (%)	Yes	15.23	<0.001,*	48.53 (±4.22)	61.52 (± 5.03)	23.89 (± 5.96)	28.43 (± 8.29)
**Behavioural**	RESTIN (%)	No	8.889	0.031,*	9.73 (± 2.52)	14.27 (± 8.52)	51.46 (± 3.26)	21.05 (± 7.03)
**Movement**	Foraging range (km)	No	7.594	0.055,*	463.6 (± 188.35)	703.02 (± 333.58)	161.48 (± 101.87)	307.05 (± 328.59)
**Movement**	Maximum speed (m s^-1^)	No	6.997	0.072,*	26.57 (± 2.03)	42.36 (± 25.72)	54.44 (± 26.02)	25.09 (± 7.54)
**Behavioural**	INTSER (%)	Yes	2.97	0.1,**	30.73 (± 5.13)	16.99 (± 1.81)	14.48 (± 5.22)	40.73 (± 6.67)
**Energy**	Daily energy expenditure (kJ day^-1^)	Yes	2.49	0.13,**	3.88 (± 0.36)	3.6 (± 0.55)	3.58 (± 0.23)	3.51 (± 0.42)
**Energy**	Daily net energy gain (kJ day^-1^)	Yes	2.09	0.17,**	5.06 (± 3.94)	6.59 (± 6.27)	1.47 (± 2.3)	10.57 (± 7.05)
**Movement**	Trip duration (h)	Yes	1.84	0.19,**	53 (± 19.64)	40.05 (± 0.06)	18.53 (± 0.23)	38.9 (± 12.84)
**Energy**	Daily energy gain (kJ day^-1^)	Yes	1.88	0.19,**	13.41 (± 5.7)	15.28 (± 9.08)	7.58 (± 3.79)	21.11 (± 10.22)
**Behavioural**	EXTSER (%)	No	4.269	0.23, NS	11.01 (± 1.47)	7.22 (± 3.23)	10.17 (± 4)	9.79 (± 6.67)
**Energy**	Mean prey weight (g)	No	3.126	0.37, NS	337.29 (± 139.77)	466.56 (± 340)	177.86 (± 61.01)	332.44 (± 301.28)
**Energy**	Number of prey per day	No	3.076	0.38, NS	0.01 (± 0.01)	0.01 (± 0.01)	0.01 (± 0.01)	0.02 (± 0.01)
**Energy**	Foraging efficiency	No	2.573	0.46, NS	2.34 (± 2.01)	8.72 (± 12.26)	1.18 (± 0.12)	4.97 (± 3.09)
**Energy**	Sd of prey weight (g)	No	2.436	0.48, NS	334.36 (± 146.75)	389.19 (± 283.87)	157.39 (± 137.42)	540.31 (± 573.91)
**Energy**	Mass at departure (kg)	Yes	0.22	0.64, NS	9.4 (± 0.93)	8.95 (± 1.04)	9.3 (± 0.28)	9.08 (± 1)

An ANOVA (when normally distributed) or a non-parametric Kruskal-Wallis test was applied (indicated by Normality column: yes or no) for selecting the most significant parameters (behavioural, movement and energetic) characterising clusters. Significance was set at P < 0.1 (*) and marginal significance to P < 0.2 (**), whereas higher values were not significant (NS). FII: foraging-in-flight. SAW: sit-and-wait. RES: resting.

**Figure 6:**
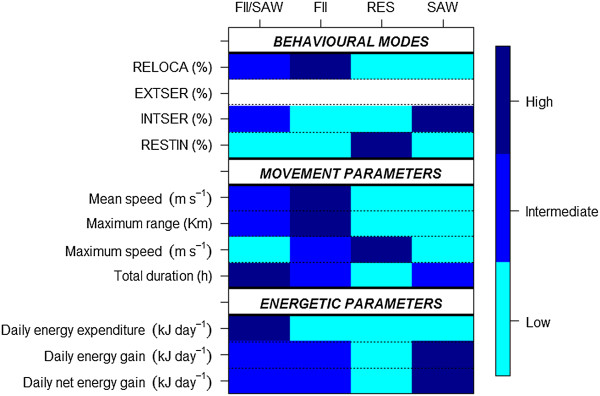
**Characterisation of foraging strategies by means of percentage of behavioural modes, movement parameters and energetic parameters.** The percentage of extensive search had no effect on cluster identification (Table 2) and we did not consider it meaningful for further interpretation (note the null extensive search values). FII: foraging-in-flight. SAW: sit-and-wait. RES: resting. RELOCA: relocation. EXTSER: extensive search. INTSER: intensive search. RESTIN: resting.

Clustering output is illustrated in Figure 6 showing mean values of behavioural modes, movement and energetic parameters. In terms of proportion of behavioural modes, Cluster 2 was represented by high relocation values (mean ± SD: 61.52% ± 5.03), as well as low intensive search (mean ± SD: 16.99% ± 1.81), and resting values (mean ± SD: 14.27% ± 8.52), and, in turn, the four individuals included in this cluster showed mainly FII strategy. This strategy was characterised by individuals travelling further away from the colony (high foraging range values) while travelling quickly, which provided low daily energy expenditure rates with intermediate both daily energy gain and net energy gain. Cluster 1 was represented by intermediate relocation (mean ± SD: 48.53% ± 4.22) and intensive search (mean ± SD: 30.73% ± 5.13) values and low resting (mean ± SD: 9.73% ± 2.52) values and, in turn, the six individuals included in this cluster switched among FII and SAW strategies. This cluster was characterised by individuals performing long trips (in duration), with intermediate foraging ranges, mean speed and low maximum speed values. In energetic terms, individuals expended more energy with intermediate rates of energy gain and net energy gain. Cluster 4 was characterised by high intensive search values (mean ± SD: 40.73% ± 6.67), as well as low values of relocation (mean ± SD: 28.43% ± 8.29), and resting (mean ± SD: 21.05% ± 7.03), and, in turn, the six individuals included in this cluster showed SAW. Individuals travelled short distances but obtained high energetic income such as daily energy gain and net energy gain. Finally, Cluster 3 was characterised by high values of resting (mean ± SD: 51.46% ± 3.26), as well as low values of both relocation (mean ± SD: 23.89% ± 5.96) and intensive search (mean ± SD: 14.48% ± 5.22). The two individuals could be mainly resting (RES) but due to the small sample size we did not consider it meaningful for further interpretation (e.g., note maximum speed for foraging trips within the cluster, Figure 6). By comparing the net energy gain of all clusters, individuals performing mainly SAW obtained a higher net energetic gain during the brooding period (marginally significant) (Table 2).

## Discussion

### A step forward in understanding foraging ecology: coupling energy-budget models and behavioural mode analysis

Following optimal foraging theory predictions [1], sit-and-wait foraging strategy obtained a higher net energetic gain during the brooding period of wandering albatrosses when they perform short foraging trips. One limitation of our approach was that we were not able to provide different heart rate values for resting and intensive search (e.g. while trying to locate, secure and swallow prey) behavioural modes. We do not consider that this caveat invalidates our results, since (1) this approach underestimates energy expenditure equally across all individuals and (2) we were interested in relative energy expenditure values and not absolute values. Thus, we consider our conclusion that sit-and-wait foraging strategy is an alternative optimal foraging strategy during brooding is still valid.

Previous studies have identified foraging-in-flight as the most optimal foraging strategy for wandering albatrosses performing long foraging trips during the incubation period [24]. During these trips, they perform large-scale movement using dynamic soaring to travel with favourable prevailing wind fields at low energy expenditure values [13]. During brooding, adults not only need to guard their young (as during incubation incubate their egg) but also need to provision chicks frequently; energetic requirements are higher than during incubation [17, 47]. During brooding, albatrosses foraging movements are limited in space and time [30], and incur body mass loss by both sexes during the 30 days of this period [28]. Due to the need to remain close to the colony in order to provision and brood the chick regularly, we hypothesise that birds cannot use their optimal foraging strategy of long distance movements (foraging-in-flight) following regional weather patterns [13]. Alternatively, wandering albatrosses need to use other foraging strategies than foraging-in-flight to cope with breeding and environmental constraints. Within this context, our results suggest that sit-and-wait might be one of these alternative foraging strategies and energetically more efficient during the brooding period.

Overall, we suggest that changes in energetic constraints of reproduction over the breeding season imposed by central place foraging can shape the use of different foraging strategies depending on the breeding stage. Additional external factors such as food availability, length of day and weather conditions change throughout the year, and the effort of birds to meet their energy requirements should therefore be adjusted to these constraints [31]. In the case of wandering albatrosses, the brooding period is the most constraining period in terms of energy needs and it occurs during the period of supposed highest prey abundance (Figure 1 in [31]). During this period day length is short, and average wind speed intermediate (even if these two factors vary much less than variation in food availability does throughout the year) (Figure 1 in [31]). These conditions are not the most favourable for the foraging-in-flight strategy which is used during daylight and strongly relies on visual cues for prey capture [51, 52], in addition to great dependence on strong wind conditions for optimal flying [13]. For all these reasons, we are confident with the conclusion that the sit-and wait foraging strategy might be more profitable during the brooding period when both breeding requirements and external factors (e.g., food availability, day duration, wind speed) constrain albatrosses to a central breeding location. In the future, it would be interesting to perform similar analyses during the incubation period, to confirm that indeed foraging-in-flight is more optimal than sit-and-wait when foraging time is not as limited as during the brooding period.

### Individual variability

Our results outline the importance of considering individual variability in foraging strategies before departing into population-level generalizations. We identified the main foraging strategy used by each bird characterised by specific movement and energetic parameters, as well as secondary alternative strategies. These birds could switch foraging strategies during a foraging trip. For instance, individuals showing a foraging-in-flight strategy can also show sit-and-wait strategy depending on the time of the day (present study, [24]). At the individual level, internal factors could decide foraging destination and external factors could constrain decision making [3]. In the present study, external factors such as wind conditions were similar for all individuals but no prey landscape information was available. Thus, contrasting foraging decisions (i.e., proportion of behavioural modes) of studied birds might be related to internal factors such as those required to fulfil energetic requirements [3, 6], while pending the assessment of the importance of prey distribution and abundance. Recent studies on albatrosses have shown that individual variation can result from age or personality differences that affect foraging strategies. For example very old birds forage in different environment than younger birds [53], while individual personality affect foraging strategies [54] that can be heritable [55].

Our results support the growing acknowledgment about the role of individual behavioural flexibility to cope efficiently with finding food resources [56]. For instance, highly variable differences among individuals have been observed in movement decisions of terrestrial mammals in relation to immediate habitat selection, with some individuals responding more to internal factors rather than to external factors [57]. Regarding selection of foraging strategies, recent studies have showed that individuals can shift from one search strategy to another not only under contrasting environmental conditions but also under similar conditions during the same period of the annual cycle in successive years [56]. This study suggests that apart from environmental conditions the cognitive abilities of individuals (previous experience; [58]) could play an important role driving changes among search strategies [56]. Thus, further research is needed to disentangle the effects of internal factors such as energy levels, cognitive abilities and age in behavioural decision-making by which animals select their optimal strategy [53–56].

### Energetic modelling issues and future applications

In the present work, we compiled and condensed the scattered information on ecological energetics and gained a fine scale energy budget model for a free-ranging pelagic predator. The instantaneous energy-budget model allows for an integration of factors that affect the movement and energy expenditure at smaller temporal scales into the energetic quantification at larger temporal scales. On the one hand, the instantaneous energy-budget model improves our understanding of the ecological energetics of flying birds at sea. Only energy expenditure during flight is usually considered when analysing optimal flying pathways among pelagic seabirds in relation to wind conditions [14, 38], and the energetic costs of resting (i.e., sitting on the water), take-off, and landing are usually neglected. In fact, the most striking finding of our study is the importance of considering fine scale behavioural modes since trip energy expenditure estimations based only on flying and resting activities underestimated energy expenditure between 18.7 and 21.5%. On the other hand, we did not explicitly considered other processes of energy expenditure (e.g., somatic maintenance) that could improve our predictions of trip net energy gain and, in turn, predicted body mass change. When more detailed data would become available, our energy-budget model could be further developed.

For the precise determination of factors that affect the rate of energy expenditure of free-ranging animals, it is necessary to obtain instantaneous measurements of energy expenditure in the field [39]. Historically, energy expenditure was estimated based on non-continuous methods such as doubly labelled water [40]. Doubly labelled water can be used to estimate energy expenditure but it provides only an average estimate over the measurement period and the period of monitoring is limited to a few days [46]. Biologging advances have provided new methods (i.e., measuring heart rate or accelerometry) to estimate continuous energy expenditure [6, 40]. Continuous energy-budget models are especially interesting for free-ranging animals foraging far from their breeding grounds for long periods, since it is impossible to observe their behaviour continuously to determine activity and energy budgets by any other way [6]. Continuous energy-budget models can be useful for quantifying energy costs of different activities, seasons, individuals and populations [40] and, in turn, understand temporal changes in the energetic requirement associated to changes in the foraging strategy.

Direct energy measurements are limited in time but they are biologically relevant if we want to predict the long-term effects of climate change on free-ranging animals. Instantaneous energy-budget models have the advantage of providing quantitative estimation of instantaneous energy expenditure. Qualitative (relative) energy expenditure values have been used in migration studies to understand Cory’s shearwaters *Calonectris diomedea* transequatorial migration detours and can be crucial in defining the main seabird corridors for conservation purposes [14]. However, quantitative approaches have the advantage to link ecological energetics to life-history traits in a wider biological context. This application is an especially interesting venue for species adapting to changing environmental conditions. For instance, breeding wandering albatrosses have increased their travel rates and flight speeds and shifted their foraging range poleward over the past 20 years, in conjunction with westerly winds [9]. These authors suggested that the predicted further intensification and poleward shift of westerly winds may affect future distribution of albatrosses and other seabirds that rely on wind conditions [9]. The present energy-budget model makes it possible not only to quantitatively predict current but also future energy expenditures [59].

## Conclusions

Directly quantifying the energy needed for animal movements leads us to understand how foragers should move across the landscape in order to maximise net energy gain allowing the testing of predictions derived from optimal foraging theory [1]. By coupling energy-budget models and behavioural mode analysis, we were able (1) to identify clusters of individuals showing different proportions of each behavioural mode, (2) to characterise those clusters following previously described foraging strategies and (3) to energetically characterise each foraging strategy with the final aim of testing optimal foraging theory predictions. We acknowledge that our results are species-specific but our novel methodological approach can be widely applied to other far-ranging animals and offer new insights on the potential effects of the predicted future increase of westerly winds on the biology of pelagic habitats in the southern oceans.

## Availability of supporting data

The data used in this study have to be requested directly to Henri Weimerskirch.

## Electronic supplementary material

Additional file 1: **Wind fields and calculations.** (DOCX 24 KB)

Additional file 2: **At-sea activity patterns.** (DOCX 342 KB)

Additional file 3: **Flying cost function.** (DOCX 699 KB)

Additional file 4: **Empirical distribution of body mass difference between arrival and departure.** (DOCX 18 KB)

Additional file 5: **Fine-scale energy expenditure.** (DOCX 27 KB)

Additional file 6: **Output of Expectation Maximization Binary Clustering algorithm.** (DOCX 78 KB)

## Abbreviations

ARS: Area-restricted search
FII: Foraging-in-flight
EXTSER: Extensive search
INTSER: Intensive search
OBMC: Observed body mass change
PBMC: Predicted body mass change
RELOCA: Relocation
RES RESTIN: Resting
RES RESTIN: Resting
SAW: Sit-and-wait
TEG: Trip energy gain
TEE: Trip energy expenditure
TNEG: Trip net energy gain
TPC: Trip prey capture data
