# Supplementary material for: Coupling instantaneous energy-budget models and behavioural mode analysis to estimate optimal foraging strategy: an example with wandering albatrosses
Source: Mov Ecol. 2014 Apr 23;2(1):8. doi: 10.1186/2051-3933-2-8 (PMC4267543; doi:10.1186/2051-3933-2-8)
Supplement: Supplementary file 1 — Additional file 1: Wind fields and calculations. (DOCX 24 KB) [file 40462_2013_19_MOESM1_ESM.docx]

**Additional file 1. Wind fields and calculations**

Wind field data were extracted from the NOAA/NCDC Blended 6-hourly 0.25° Sea Surface Winds thanks to the Environmental Research Division's Data Access Program managed by the National Oceanic and Atmospheric Administration of USA (data available at <http://coastwatch.pfeg.noaa.gov/erddap/griddap/ncdcOw6hr.html>). The Blended Sea Winds contain globally gridded, high resolution ocean surface vector winds and wind stress on a global 0.25° grid, and multiple time resolutions (e.g., 6-hourly) from the 9^th^ of July 1987 to present by blending observations from multiple satellites (http://www.ncdc.noaa.gov/oa/rsad/seawinds.html). Zonal and meridional wind speed components were provided at a height of 10m above the sea level. Since wandering albatrosses fly at this height [1], we did not apply any correction to the wind speed estimation (c.f., [2]).

Each foraging trip was divided in 6-hour segments following the timing as sea surface winds and dividing the tracks in four periods: midnight to 6am, 6am to noon, noon to 6pm and 6pm to midnight. Afterwards, the nearest 6-h zonal and meridional wind components (*u* and *v*, respectively) were assigned to each position and the modulus of wind speed *w* and direction (α in degrees, ranging from 0° to 360° indicative of North-East-South-West; http://www.ncl.ucar.edu/Document/Functions/Built-in/atan2.shtml) were estimated as follows:

*w = sqrt(u^2^ + v^2^ )*

α*= 180 + atan2(u,v) * (180/π)*

**References**

1. Pennycuick CJ: **The flight of petrels and albatrosses (Procellariiformes), observed in South Georgia and its vicinity**. *Philos. Trans. R. Soc. London.Series B, Biol. Sci.* 1982, **300**:75–106.

2. Wakefield E, Phillips R, Trathan P, Arata J, Gales R, Huin N, Robertson G, Waugh S, Weimerskirch H, Matthiopoulos J: **Habitat preference, accessibility and competiton limit the global distribution of breeding black-browed albatrosses**. *Ecol. Monogr.* 2010.
