# Supplementary material for: Coupling instantaneous energy-budget models and behavioural mode analysis to estimate optimal foraging strategy: an example with wandering albatrosses
Source: Mov Ecol. 2014 Apr 23;2(1):8. doi: 10.1186/2051-3933-2-8 (PMC4267543; doi:10.1186/2051-3933-2-8)
Supplement: Supplementary file 2 — Additional file 2: At-sea activity patterns. (DOCX 342 KB) [file 40462_2013_19_MOESM2_ESM.docx]

**Additional file 2. At-sea activity patterns**

Example of (a) typical activity pattern of one wandering albatross along its foraging trip, with two specific sections at the beginning (b) and end of the foraging trip (c).The black dashed line indicates the threshold of 10 km h^-1^.

(a)

**b**

**c**

(b)

(c)
