# Supplementary material for: Coupling instantaneous energy-budget models and behavioural mode analysis to estimate optimal foraging strategy: an example with wandering albatrosses
Source: Mov Ecol. 2014 Apr 23;2(1):8. doi: 10.1186/2051-3933-2-8 (PMC4267543; doi:10.1186/2051-3933-2-8)
Supplement: Supplementary file 3 — Additional file 3: Flying cost function. (DOCX 699 KB) [file 40462_2013_19_MOESM3_ESM.docx]

**Additional file 3. Flying cost function**

The effect of wind speed *w* on energy expenditure cannot be neglected since wind speed can have important implications for the energy budget during flying activities [1, 2]. [3] provided an instantaneous index of energy expenditure (e.g., heart rate) values but they did not consider the effect wind speed *w* (only the angle between flight and wind direction, *θ*). Thus, we adapted the flying cost function developed by [1], which considered both the effect of *θ* and *w,* to wandering albatrosses using field data from [3]. We were interested in extracting the relationship of energy expenditure with *θ* and *w*, but also adapting the arbitrary cost units of this study to real heart rate units (beats min^-1^) (*sensu* [3]). Different steps were necessary for that:

(1) We estimated the cost function developed in [1] as a polynomial expression and represented in the figure below:

*Cost = 30 +* 2.381e-09 ** θ +* -9.667e-01 ** w +*1.093e-02 ** θ * w*

(2) [3] provided a mean (± SD) heart rate value for angles between 0º and 180º (at intervals of 30º) (Figure 3b in [3]). We assumed that this relationship was an average picture of the relationship which might integrate all wind speed *w* values. For that we compared the effect of *θ* on heart rate (i.e., energy expenditure) in [3] and [1] and both showed a linear increasing trend of flying cost with respect to *θ*, showing a similar slope in both cases (mean (±SE) for [3]: 0.169 (± 0.009); for [1]: 0.164 ± 0.008):


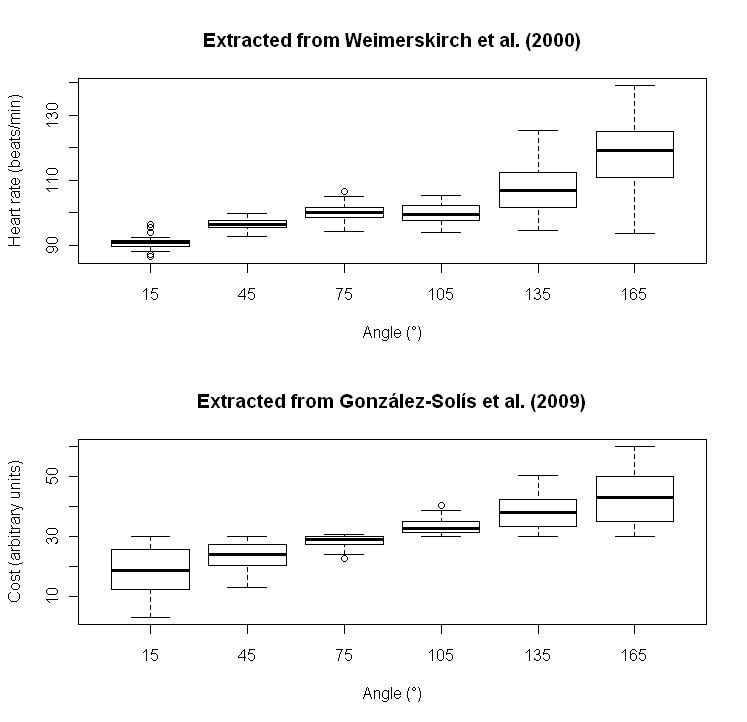


Thus, we were confident that patterns of flying costs were similar in both studies.

(3) Given the difference in the range of cost values and units of both studies, we estimated real heart rate values based on arbitrary cost units by linear regression. We found the following relationship:

*Heart rate (beats min^-1^) = 80.085 + 1.153* cost (arbitrary units)*

(4) By combining previous two equations, we were able to estimate heart rate units for all the combinations of angles *θ* and wind speeds *w*. The resulting flying cost function is represented in the Figure 1b.

To better adjust heart rate values for flying modes F – J (i.e., directly after take-off; Figure 1) to reality, we introduced correction factors based on [3] that reported that heart rate values of flying albatrosses decreased after two hours of take-off to basal values. We assumed that the flying cost function estimates energy expenditure in optimal conditions (i.e., at least 2 hours of flight, [3]). Thus, previous heart rate values for flying activities should be higher (see the decreasing trend of filled triangles of activities F-J in Figure 1a). Taking the heart rate value of mode J (i.e., 720 min after take-off) as reference, we computed the correction factors to be 1.39, 1.19, 1.13 and 1.09 for flying positions 10, 30, 60 and 120min after take-off, respectively. The flying cost function was applied to flying activities (F to J in Figure 1a) in order to obtain energy expenditure values.

**References**

1. González-Solís J, Felícisimo A, Fox J, Afanasyev V, Kolbeinsson Y, Muñoz J: **Influence of sea surface winds on shearwater migration detours**. *Mar. Ecol. Prog. Ser.* 2009, **391**:221–230.

2. Raymond B, Shaffer SA, Sokolov S, Woehler EJ, Costa DP, Einoder L, Hindell M, Hosie G, Pinkerton M, Sagar PM, Scott D, Smith A, Thomson DR, Vertigan C, Weimerskirch H: **Winds and prey availability determine shearwater foraging distribution in the Southern Ocean**. *PLoS One* 2010, **5**:e109.

3. Weimerskirch H, Guionnet T, Martin J, Shaffer SA, Costa DP: **Fast and fuel efficient? Optimal use of wind by flying albatrosses**. *Proc. R. Soc. B Biol. Sci.* 2000, **267**:1869–1874.
