# Supplementary material for: Coupling instantaneous energy-budget models and behavioural mode analysis to estimate optimal foraging strategy: an example with wandering albatrosses
Source: Mov Ecol. 2014 Apr 23;2(1):8. doi: 10.1186/2051-3933-2-8 (PMC4267543; doi:10.1186/2051-3933-2-8)
Supplement: Supplementary file 4 — Additional file 4: Empirical distribution of body mass difference between arrival and departure. (DOCX 18 KB) [file 40462_2013_19_MOESM4_ESM.docx]

**Additional file 4. Empirical distribution of body mass difference between arrival and departure**

We extracted information on the empirical distribution of body mass difference between arrival and departure from the long-term database of wandering albatross tracking data which extended from 1989 to 2010 [1]. Body mass was not measured in all cases and we were able to compile information for 97 individuals of known sex. Since same individuals were equipped twice during the 20-year period, we randomly selected one measure per bird. Moreover, birds were equipped with satellite tracking devices during two different breeding stages: incubation and brooding. Since the energetic contribution to breeding for both sexes and breeding stages are different [2], we tested the effect of sex, stage, additive models and their statistical interaction. Models were ranked based on their Akaike Information Criteria corrected for small sample sizes (AICc) values. The model with the lowest AICs is considered the best compromise between model deviance and model complexity, i.e. the number of parameters in the model. When models are within 4 points of AICs, they are considered statistically equivalent and the model with fewest parameters was chosen. This approach yielded that the main factor explaining observed mass difference patterns was the breeding stage, followed by the additive and interaction effects of stage and sex (Table S1).

Table S1.Modelling the body mass difference between arrival and departure from the long-term database of wandering albatross tracking data depending on the sex, stage, additive models and their statistical interactions. Models were ranked based on their Akaike Information Criteria corrected (AICc) for small sample sizes (AICc) values. ΔAICc = AICc – min(AICc).

| **Models** | **Number of parameters** | **AICc** | **ΔAICc** |
| --- | --- | --- | --- |
| Stage | 3.000 | 217.563 | 0.000 |
| Sex + Stage | 4.000 | 219.542 | 1.979 |
| Sex * Stage | 5.000 | 221.766 | 4.203 |
| Null | 2.000 | 225.741 | 8.178 |
| Sex | 3.000 | 227.845 | 10.282 |

**References**

1. Weimerskirch H, Louzao M, de Grissac S, Delord K: **Changes in wind pattern alter albatross distribution and life-history traits**. *Science* 2012, **335**:211–214.

2. Salamolard M, Weimerskirch H: **Relationship between foraging effort and energy requirement throughout the breeding season in wandering albatross**. *Funct. Ecol.* 1993, **7**:643–652.
