# Supplementary material for: Coupling instantaneous energy-budget models and behavioural mode analysis to estimate optimal foraging strategy: an example with wandering albatrosses
Source: Mov Ecol. 2014 Apr 23;2(1):8. doi: 10.1186/2051-3933-2-8 (PMC4267543; doi:10.1186/2051-3933-2-8)
Supplement: Supplementary file 5 — Additional file 5: Fine-scale energy expenditure. (DOCX 27 KB) [file 40462_2013_19_MOESM5_ESM.docx]

**Additional file 5. Fine-scale energy expenditure**

Figure characterising fine-scale energy expenditure of albatross activity (mean and CI 95%). A: ‘landing’; B: ’30 min after landing’; C: ‘resting’; D: ’30 min before take-off’; E: ‘take-off’; F, G, H, I and J correspond to ‘flying’ 10, 30, 60, 120 and 720 min after take-off’, respectively.

*
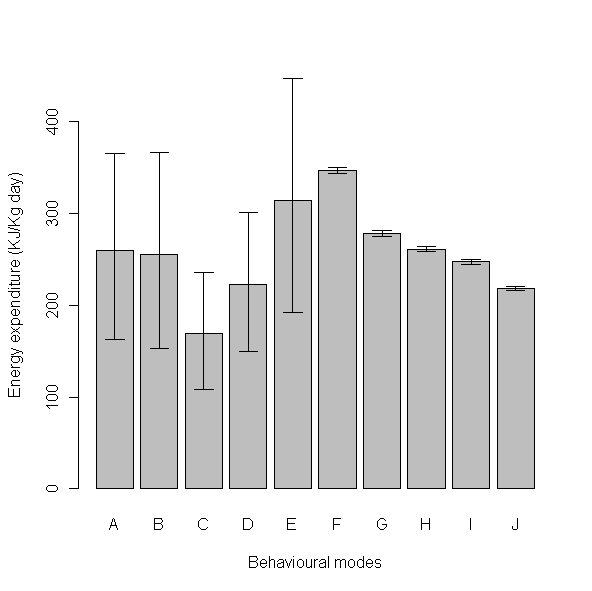
*
