# Supplementary material for: Coupling instantaneous energy-budget models and behavioural mode analysis to estimate optimal foraging strategy: an example with wandering albatrosses
Source: Mov Ecol. 2014 Apr 23;2(1):8. doi: 10.1186/2051-3933-2-8 (PMC4267543; doi:10.1186/2051-3933-2-8)
Supplement: Supplementary file 6 — Additional file 6: Output of Expectation Maximization Binary Clustering algorithm. (DOCX 78 KB) [file 40462_2013_19_MOESM6_ESM.docx]

**Additional file 6. Output of Expectation Maximization Binary Clustering algorithm**

Figure describing the output of the Expectation Maximization Binary Clustering (EMBC) algorithm showing the four regions of high and low values for speed/turn values characterising behavioural modes (EXTSER: extensive search, RELOCA: relocation, INTSER: intensive search, RESTIN: resting on the water). Time series of (b) speed and (c) turning angle showing the mass of captured prey (size of green circles is proportional to the prey mass), as well as the instantaneous behavioural modes. Grey shadow indicates night periods, whereas dotted horizontal line in travel speeds figures indicating the threshold of 2.7 m s^-1^[1].

(a)


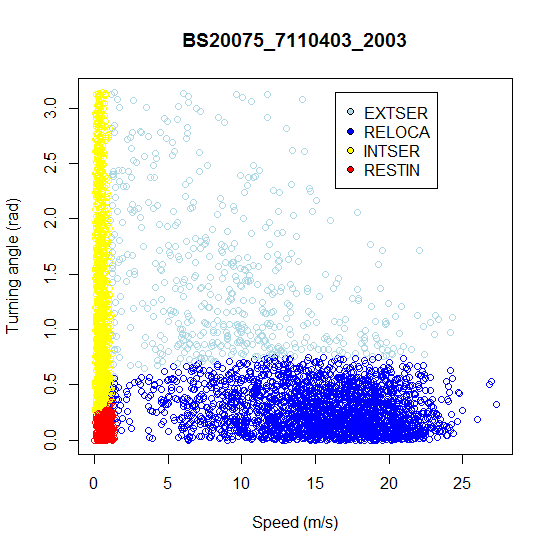


(b)


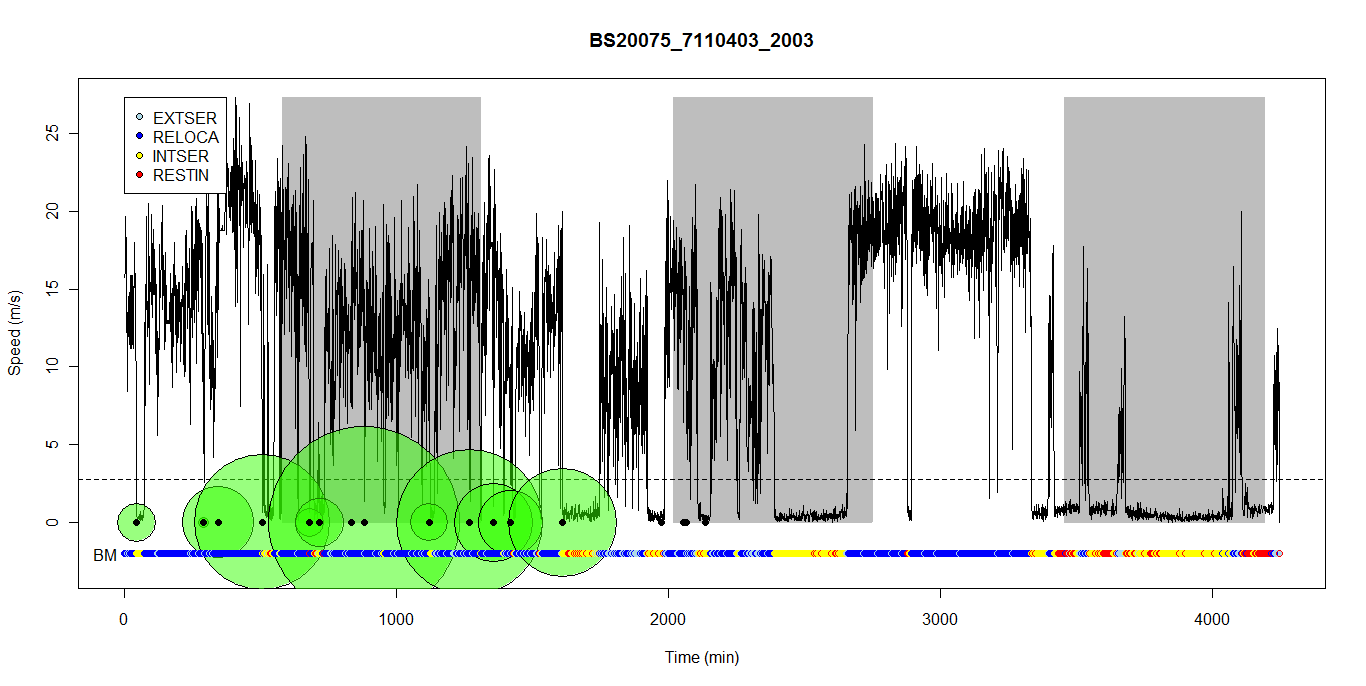


(c)


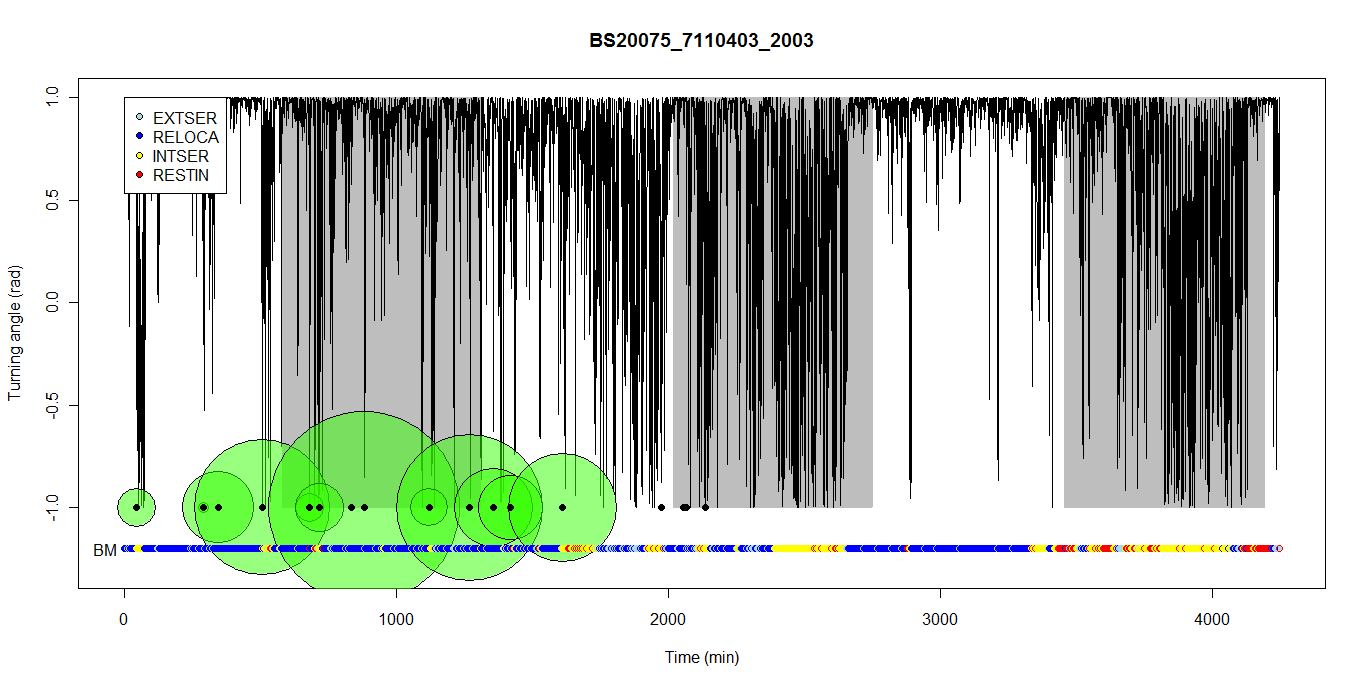


**References**

1. Weimerskirch H, Bonadonna F, Bailleul F, Mabille G, Dell’Omo G, Lipp HP: **GPS tracking of foraging albatrosses**. *Science* 2002, **295**:1259.
